# Kidney outcomes of malignant hypertension-associated thrombotic microangiopathy in patients with and without IgA nephropathy: a propensity score-matched analysis

**DOI:** 10.1093/ckj/sfaf017

**Published:** 2025-02-21

**Authors:** Wenchuan Li, Rong Lian, Yuejiao Li, Xingji Lian, Zefang Dai, Zhong Zhong, Wanxin Shi, Yiqin Wang, Wei Chen, Jianbo Li, Feng He

**Affiliations:** Department of Nephrology, Guangzhou First People's Hospital, The Second Affiliated Hospital, school of medicine, South China University of Technology, Guangzhou, China; Department of Nephrology, Guangzhou First People's Hospital, The Second Affiliated Hospital, school of medicine, South China University of Technology, Guangzhou, China; Department of Nephrology, Guangzhou First People's Hospital, The Second Affiliated Hospital, school of medicine, South China University of Technology, Guangzhou, China; Department of Nephrology, Guangzhou First People's Hospital, The Second Affiliated Hospital, school of medicine, South China University of Technology, Guangzhou, China; Department of Geriatrics, Guangzhou First People's Hospital, The Second Affiliated Hospital, School of Medicine, South China University of Technology, Guangzhou, China; Department of Nephrology, The First Affiliated Hospital, Sun Yat-sen University, NHC Key Laboratory of Clinical Nephrology (Sun Yat-sen University) and Guangdong Provincial Key Laboratory of Nephrology, Guangzhou, China; Department of Nephrology, The First Affiliated Hospital, Sun Yat-sen University, NHC Key Laboratory of Clinical Nephrology (Sun Yat-sen University) and Guangdong Provincial Key Laboratory of Nephrology, Guangzhou, China; Department of Nephrology, Guangzhou First People's Hospital, The Second Affiliated Hospital, school of medicine, South China University of Technology, Guangzhou, China; Department of Nephrology, The First Affiliated Hospital, Sun Yat-sen University, NHC Key Laboratory of Clinical Nephrology (Sun Yat-sen University) and Guangdong Provincial Key Laboratory of Nephrology, Guangzhou, China; Department of Nephrology, The First Affiliated Hospital, Sun Yat-sen University, NHC Key Laboratory of Clinical Nephrology (Sun Yat-sen University) and Guangdong Provincial Key Laboratory of Nephrology, Guangzhou, China; Department of Nephrology, The First Affiliated Hospital, Sun Yat-sen University, NHC Key Laboratory of Clinical Nephrology (Sun Yat-sen University) and Guangdong Provincial Key Laboratory of Nephrology, Guangzhou, China; Department of Nephrology, Guangzhou First People's Hospital, The Second Affiliated Hospital, school of medicine, South China University of Technology, Guangzhou, China

**Keywords:** IgA nephropathy, kidney biopsy, kidney replacement therapy, malignant hypertension, thrombotic microangiopathy

## Abstract

**Background:**

IgA nephropathy (IgAN) can cause hypertension, and severe hypertension can exacerbate the progression of IgAN. However, the long-term kidney outcome of malignant hypertension (mHTN)-associated thrombotic microangiopathy (TMA) with IgAN is not well defined.

**Methods:**

A total of 292 individuals with mHTN-associated TMA confirmed by kidney biopsy were included. Propensity score matching (PSM) analysis was performed to adjust for clinical characteristics in the comparison between cases with and without IgAN. Cox regression analysis was utilized to identify risk factors associated with long-term kidney outcome.

**Results:**

A total of 86 mHTN-associated TMA with IgAN patients were compared with 206 mHTN-associated TMA with non-IgAN patients. After PSM, 61 pairs of patients with mHTN-associated TMA were matched. The mHTN-associated TMA with IgAN patients exhibited significantly lower serum albumin, higher 24-hour proteinuria, and a higher ratio of global sclerosis than those with non-IgAN. mHTN-associated TMA with IgAN was independently associated with impaired kidney function recovery [hazard ratio (HR), 0.48; 95% confidence interval (CI), 0.24–0.96, *P* = .038] compared with non-IgAN. This association remained significant after PSM (HR, 0.41; 95% CI, 0.17–0.99, *P* = .047). In addition, mHTN-associated TMA with IgAN was independently associated with kidney replacement therapy (KRT) compared with non-IgAN (HR, 2.31; 95% CI, 1.38–3.88; *P* = .002). This difference remained significant after PSM comparison (HR, 2.38; 95%CI, 1.14–4.99; *P* = .021). In addition, mHTN-associated TMA with IgAN patients had a higher incidence of receiving KRT and a lower incidence of kidney function recovery with a 25% reduction in creatinine levels than in non-IgAN patients, regardless of intensive blood pressure control.

**Conclusions:**

The long-term kidney outcomes for mHTN-associated TMA patients with concomitant IgAN are significantly poorer than that of patients with non-IgAN. Monitoring kidney pathological characteristics will aid management and risk assessment at an early stage.

KEY LEARNING POINTS **What was known:**

- IgA nephropathy (IgAN) can lead to hypertension, which can worsen IgAN progression.
- Malignant hypertension (mHTN)-associated thrombotic microangiopathy (TMA) is linked to poor kidney outcomes.
- The long-term kidney prognosis of mHTN-associated TMA with IgAN was previously undefined.

**This study adds:**

- mHTN-associated TMA patients with IgAN have poorer kidney outcomes than those without IgAN.
- IgAN is an independent risk factor for deteriorated kidney function and kidney replacement therapy in mHTN-associated TMA.
- Kidney pathological characteristics are crucial for early management and risk assessment in mHTN-associated TMA with IgAN.

**Potential impact:**

- Early identification of IgAN in mHTN-associated TMA can guide more aggressive treatment strategies.
- Monitoring Kidney pathological features can improve patient prognosis in mHTN-associated TMA.
- This study's findings may influence clinical guidelines for managing mHTN-associated TMA with IgAN.

## INTRODUCTION

Malignant hypertension (mHTN) is a severe hypertensive emergency characterized by rapid elevation of blood pressure and acute organ damage [1–3]. The incidence of mHTN is higher in males, smokers, and those with a history of kidney artery stenosis [4]. Without prompt treatment, mHTN can cause permanent damage to the central nervous system, cardiovascular system, and kidney [3]. Thrombotic microangiopathy (TMA) is a complication attributed to mHTN, which manifests as diffuse capillary loop wrinkling and capsule thickening, marked kidney artery intimal thickening, vessel wall thickening with an ‘onion-peel’ appearance, fibrinoid necrosis, intravascular thrombosis, and so on [5–7]. This may result in end-organ ischemia and infarction, mostly commonly affecting the brain and kidney [8, 9]. However, the risk factors for kidney prognosis in patients with mHTN-associated TMA are still unclear.

IgA nephropathy (IgAN), a major cause of primary glomerulonephritis, is closely associated with mHTN. First, IgAN can precipitate the development of hypertension. The kidney plays a crucial role in the regulation of blood pressure. In patients with IgA nephropathy, the deposition of immune complexes in the mesangial areas of the glomeruli leads to local inflammation and kidney damage [10, 11]. Kidney damage leads to sodium retention and activation of the renin-angiotensin-aldosterone system, resulting in renal hypertension. Second, severe hypertension in the context of mHTN causes endothelial dysfunction, and activation of the coagulation cascade can lead to the formation of microthrombi. These can impede blood flow in the kidney microvasculature and exacerbate kidney injury, which can deteriorate kidney function [12, 13]. It is unclear whether mHTN-associated TMA patients with concomitant IgAN have a poor kidney prognosis.

Therefore, this study investigates the clinicopathological characteristics of patients with mHTN-associated TMA, and identifies risk factors that affect kidney prognosis. This study highlights the prognostic significance of concomitant IgAN in mHTN-associated TMA patients and guides the clinical management of mHTN patients.

## MATERIALS AND METHODS

### Study population and cohort

This prospective study enrolled patients who underwent clinical kidney biopsy at the First Affiliated Hospital of Sun Yat-sen University from January 2008 to June 2023 and were subsequently pathologically diagnosed with mHTN-associated TMA. Patients with <3 months of follow-up were excluded from the study. Patients were categorized according to the presence or absence of IgA nephropathy as a comorbidity, and the study cohort was divided into two distinct groups: an mHTN-associated TMA with IgAN group (IgAN, *n* = 86) and an mHTN-associated TMA without IgAN group (non-IgAN, *n* = 206). To ensure comparability and minimize selection bias, we applied propensity score matching (PSM) to adjust for baseline differences between the non-IgAN and IgAN groups. This study was conducted in accordance with the ethical standards of the Declaration of Helsinki. This study was approved by The First Affiliated Hospital of Sun Yat-sen University Institutional Review Boards, Guangzhou, China [No. IEC (2022) 710]. Written informed consent was obtained from all patients. No financial compensation was provided.

### Definitions

Malignant hypertension is defined as a clinical syndrome of accelerated hypertension with target organ damage, primarily characterized by a rapid rise in diastolic blood pressure (DBP) >130 mmHg [14]. This condition is often accompanied by hypertensive retinopathy, which includes signs such as retinal hemorrhages, exudates, and optic disc edema [14]. The diagnosis of mHTN with TMA was confirmed based on kidney pathological features, including various pathological changes such as capillary loop wrinkling, capsule thickening, significant kidney artery intimal thickening, vessel wall “onion-peel” thickening, fibrinoid necrosis, intravascular thrombosis, ischemic glomerular alterations, and tubular necrosis [5, 6]. IgAN, diagnosed based on kidney biopsy pathology, refers to a condition in which immunoglobulin A (IgA) accumulates in the kidneys, causing inflammation and potentially progressive kidney damage [15]. In our study, an average systolic blood pressure (SBP) ≤130 mmHg in the 3 days before hospital discharge was used as the criterion for intensive blood pressure management, based on consultation with both international and national clinical guidelines [16, 17].

### Data collection and kidney histopathology

Blood and urine samples were collected within the first 24 hours on admission, and all patients underwent chest radiography, kidney ultrasound, and echocardiography. Baseline clinical and demographic data were collected, including age, sex, body mass index (BMI), blood pressure measurements, history of hypertension, daily urine protein excretion, hemoglobin, albumin, platelet count, cholesterol, triglyceride, low-density lipoprotein (LDL-c), high-density lipoprotein (HDL-c), urea nitrogen, serum creatinine, uric acid, and serum complement C3 and C4. The estimated glomerular filtration rate (eGFR) was calculated using the Chronic Kidney Disease Epidemiology Collaboration (CKD-EPI) equation [18]. Mean arterial pressure was defined as one-third of SBP plus two-thirds of DBP. Prescription data included statins, beraprost sodium, sulodexide, sacubitril/valsartan, angiotensin-converting enzyme inhibitor (ACEI)/angiotensin II receptor blockers (ARBs), and other antihypertensive drugs (including α-blockers, β-blockers, calcium channel blockers).

Percutaneous kidney biopsy specimens were routinely processed according to standard protocols. Kidney biopsies were processed for routine light microscopy, electron microscopy, and direct immunofluorescence using fluorescein isothiocyanate-conjugated antibodies specific for human IgG, IgM, IgA, C1q, C3, and κ and λ light chains. All kidney biopsy results were assessed by two senior pathologists. In cases of disagreement, a nephrologist participated in further discussions until a resolution was reached. The number and ratio of glomeruli, global sclerosis and segmental sclerosis were collected from the pathology reports. Tubulointerstitial parameters were also recorded, including interstitial fibrosis and tubular atrophy, and tubular epithelial cell sloughing. In addition, vascular parameters were recorded, including arteriolar hyalinosis, fibrinoid necrosis, onion skin lesions, intravascular thrombosis, and intravascular erythrocyte fragments. Electron microscopic evaluation identified subendothelial and mesangial deposits and assessed the degree of endothelial cell swelling.

### Study outcomes

The primary outcome was recovery of kidney function, which was defined as a >50% decrease in serum creatinine from baseline, a decrease in serum creatinine to normal levels (based on the normal reference range of <1.3 mg/dl established by the hospital), or kidney survival free from hemodialysis or peritoneal dialysis for at least 1 month for dialysis-dependent patients. The secondary outcome of this study was kidney replacement therapy (KRT), which was defined as the need for hemodialysis, peritoneal dialysis, or kidney transplantation during follow-up. All patients were followed up by nephrologists and trained nurses through office visits or telephone interviews, and the last follow-up date was 30 June 2023.

### Statistical analysis

Continuous variables were presented as the mean and standard deviation (SD) for normally distributed data, and as the median with interquartile range (IQR) for non-normally distributed data. Categorical variables were presented as frequencies (percentages). Continuous variables were compared using the Mann–Whitney *U*-test for non-normally distributed variables and the Student's *t*-test for normally distributed variables. Categorical variables were expressed as frequencies (percentages), and analyzed with the chi-squared test or Fisher exact test to compare group differences between the non-IgAN and IgAN groups. Time to study outcome was estimated using the Kaplan–Meier model, with survival comparisons between the non-IgAN and IgAN groups based on the log-rank test. The adjusted hazard ratios (HRs) with 95% confidence intervals (CIs) were estimated using univariate and multivariate Cox regression analyses to identify variables associated with kidney outcomes in patients with mHTN-associated TMA. To adjust for the baseline differences and to minimize potential selection bias, we applied PSM between the non-IgAN and IgAN groups. A 1:1 match was performed using the greedy-matching algorithm with a 0.02 caliper [19, 20]. Survival analysis was used to assess the prognosis before and after PSM. All analyses were considered statistically significant if the two-tailed *P* value was <0.05. All statistical analyses were performed using SPSS (version 23.0; IBM, Armonk, NY, USA).

## RESULTS

### Baseline demographics and characteristics

Our final cohort consisted of 292 patients [mean (SD) age, 35.74 (8.66) years] were available for evaluation and were pathologically diagnosed with mHTN-associated TMA, with males constituting 89.0%. A flowchart illustrating this process was presented in Supplementary Fig. S1. Baseline characteristics of patients before and after PSM were shown in Table 1. A total of 206 (70.5%) patients were initially assigned to the non-IgAN group, while 86 (29.5%) patients were assigned to the IgAN group. Compared to non-IgAN patients, IgAN patients were younger, had lower levels of BMI, blood platelet count, serum albumin, and serum complement C3 concentrations, but had higher levels of serum creatinine and 24-hour proteinuria. In addition, the non-IgAN patients were more likely to be treated with α-blockers and beraprost sodium treatment.

**Table 1:** Baseline characteristics of patients.

		Entire cohort			Propensity score-matched cohort		
Characteristic	Total (*n* = 292)	Non-IgAN (*n* = 206)	IgAN (*n* = 86)	*P* value	Non-IgAN (*n* = 61)	IgAN (*n* = 61)	*P* value
Demographics							
Age, mean (SD), y	35.74 (8.66)	37.36 (8.26)	31.85 (8.390)	<.001	34.13 (6.19)	34.48 (8.17)	.794
Male, *n* (%)	260 (89.0)	187 (90.8)	73 (84.9)	.142	54 (88.5)	54 (88.5)	1.000
BMI, kg/m^2^	24.78 (4.02)	25.48 (4.13)	23.08 (3.02)	<.001	24.53 (3.99)	24.05 (3.11)	.455
Smoking, *n* (%)	129 (44.2)	104 (50.5)	25 (29.1)	.001	21 (34.4)	24 (39.3)	.573
Drinking, n (%)	84 (28.8)	66 (32.0)	18 (20.9)	.056	15 (24.6)	17 (27.9)	.681
Baseline BP status, mean (SD), mmHg							
SBP	160.89 (29.29)	161.04 (31.17)	160.52 (24.34)	.880	166.02 (32.82)	161.28 (26.31)	.381
DBP	101.43 (21.39)	100.68 (22.28)	103.24 (19.09)	.351	105.16 (23.46)	102.80 (20.03)	.551
MAP	121.26 (22.93)	120.81 (24.07)	122.35 (20.03)	.601	125.43 (25.66)	122.31 (21.32)	.467
Laboratory values, mean (SD)							
Hemoglobin, g/l	106.59 (22.89)	107.98 (21.64)	103.27 (25.46)	.135	106.59 (20.76)	103.80 (26.57)	.520
Blood platelets count, 10^9^/l	263.63 (87.24)	271.08 (91.76)	245.80 (72.75)	.013	251.33 (89.48)	250.67 (79.660)	.966
Serum albumin, g/l	36.79 (4.87)	37.57 (4.34)	35.51 (5.71)	.003	38.20 (4.19)	35.69 (5.97)	.008
Total cholesterol, mmol/l	4.91 (1.37)	4.85 (1.34)	5.04 (1.43)	.272	5.01 (1.48)	4.98 (1.42)	.934
Triglyceride, median (IQR), mmol/l	1.77 (1.31, 2.32)	1.81 (1.31, 2.32)	1.69 (1.31, 2.31)	.686	1.91 (1.38, 2.24)	1.77 (1.30, 2.42)	.539
LDL-C, mmol/l	3.05 (0.99)	3.04 (0.96)	3.07 (1.08)	.776	3.06 (0.92)	3.06 (1.10)	.998
HDL-C, mmol/l	1.09 (0.54)	1.06 (0.49)	1.16 (0.64)	.141	1.06 (0.66)	1.10 (0.50)	.693
Urea nitrogen, median (IQR), mmol/l	15.0 (10.8, 19.9)	14.95 (10.50, 19.70)	15.20 (11.60, 21.30)	.108	15.1 (11.10, 19.25)	14.80 (11.45, 20.95)	.607
Serum creatinine, mg/dl	526.59 (326.20)	492.93 (287.85)	607.22 (393.85)	.016	531.72 (310.10)	601.61 (417.22)	.296
eGFR, median (IQR), ml/min/1.73 m^2^	10.32 (6.32, 19.59)	10.73 (6.83, 20.33)	9.76 (4.96, 18.84)	.150	10.97 (6.25, 20.32)	10.15 (4.59, 21.41)	.216
24-h proteinuria, median (IQR), g/day	1.41 (0.84, 2.55)	1.26 (0.70, 2.08)	2.09 (1.09, 3.49)	<.001	1.20 (0.62, 1.94)	2.02 (1.10, 3.42)	<.001
Complement C3, g/l	1.00 (0.0.23)	1.03 (0.24)	0.93 (0.18)	<.001	0.98 (0.24)	0.96 (0.19)	.688
Complement C4, g/l	0.31 (0.11)	0.31 (0.12)	0.31 (0.09)	.889	0.29 (0.13)	0.32 (0.09)	.204
Ejection fraction, %	60.67 (9.59)	60.52 (9.98)	61.00 (8.74)	.718	59.07 (10.82)	60.83 (8.82)	.370
Kidney parenchyma thickness, median (IQR), cm							
Left	1.4 (1.23, 1.60)	1.5 (1.3, 1.6)	1.3 (1.1, 1.6)	<.001	1.4 (1.2, 1.6)	1.35 (1.1, 1.6)	.194
Right	1.4 (1.20, 1.60)	1.4 (1.2, 1.6)	1.4 (1.1, 1.5)	.026	1.4 (1.2, 1.6)	1.40 (1.2, 1.6)	.824
Base medications, *n* (%)							
ACEI/ARB	175 (59.9)	126 (61.2)	49 (57.0)	.506	36 (59.0)	38 (62.3)	.711
CCB	282 (96.6)	198 (96.1)	84 (97.7)	.505	59 (96.7)	61 (100.0)	.154
β-blocker	251 (86.0)	176 (85.4)	75 (87.2)	.691	49 (80.3)	54 (88.5)	.212
α-blocker	181 (62.0)	142 (68.9)	39 (45.3)	<.001	43 (70.5)	27 (44.3)	.003
Sacubitril/valsartan	66 (22.6)	45 (21.8)	21 (24.4)	.632	5 (8.2)	16 (26.2)	.008
Beraprost sodium	75 (25.8)	60 (29.1)	15 (17.6)	.042	17 (27.9)	10 (16.7)	.139
Sulodexide	149 (51.0)	112 (54.4)	37 (43.0)	.077	29 (47.5)	27 (44.3)	.716
Febuxostat	58 (19.9)	44 (21.4)	14 (16.3)	.321	6 (9.8)	12 (19.7)	.126
Statin	133 (45.5)	97 (47.1)	36 (41.9)	.414	17 (27.9)	26 (42.6)	.088

Data are presented as median (interquartile range) or number (%).

MAP, mean arterial pressure; ITS, interventricular septum thickness; CCB, calcium channel blocker.

After PSM, 61 IgAN patients were matched with 61 non-IgAN patients (Table 1). Baseline characteristics were reassessed after PSM and showed that a satisfactory balance had been achieved between the two groups. Patients with IgAN had significantly lower serum albumin levels [mean (SD), 35.69 (5.97) vs. 38.20 (4.19), *P* = .008] and higher levels of 24-hour proteinuria [median (IQR), 2.02 (1.10, 3.42) vs. 1.20 (0.62, 1.94), *P* < .001] compared to non-IgAN patients. Regarding medical treatment, IgAN patients were more likely to be treated with sacubitril/valsartan [16 (26.2%) vs. 5 (8.2%), *P* = .008], but less likely to be treated with α-blockers treatment [27 (44.3%) vs. 43 (70.5%), *P* = .003] compared to non-IgAN patients. No differences in other medications were observed between the two groups.

### Kidney histopathological characteristics

**Figure 1:**
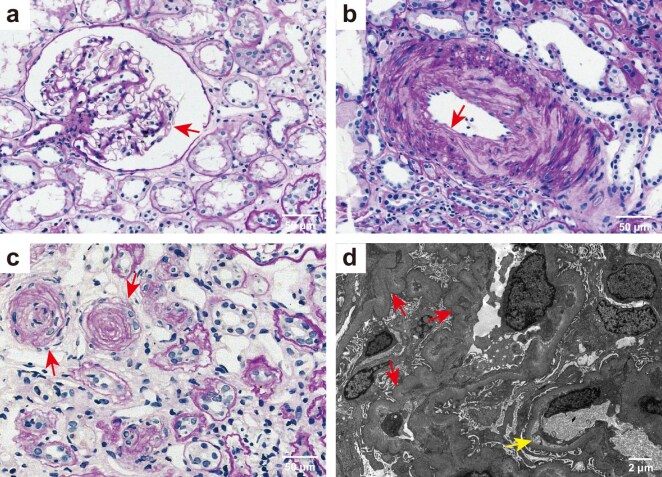
Representative light and electron microscopic findings of malignant hypertension-associated TMA (**a**) Periodic acid-Schiff (PAS) staining showing diffuse winkling of the capillary loop and capsular thickening (arrow), tubular atrophy and interstitial fibrosis (original magnification ×400). (**b**) PAS staining showing marked thickening of the medial layer in kidney arterioles (arrow) (original magnification ×400). (**c**) PAS staining showing vessel walls thickening with an onion-peel appearance and luminal narrowing and occlusion (arrow). Tubular atrophy and interstitial fibrosis are present (original magnification ×400). Scale bars 20 μm, in a–c. (**d**) Electronic micrograph showed diffuse winkling of the capillary loop (red arrow) and mild segmental subendothelial widening with flocculent material underneath (yellow arrow). Scale bar 2 μm.

Percutaneous kidney biopsy was performed on all patients with mHTN-associated TMA (Fig. 1). Light microscopic analysis revealed the characteristic pathological alterations in mHTN-associated TMA, including diffuse capillary loop twisting and capsular thickening (Fig. 1a), typical intimal thickening and mucinous degeneration of the kidney artery (Fig. 1b), and the vessel wall thickening with an onion-peel appearance (Fig. 1c). Electron microscopy showed diffuse winkling of the capillary loop and prominent subendothelial widening with flocculent material underneath (Fig. 1d).

Kidney histopathological findings of the patients were shown in Table 2. Compared to non-IgAN patients, IgAN patients had a significantly higher ratio of global sclerosis (*P* < .001) and tubular atrophy/interstitial fibrosis (*P* < .001), but had lower ratio of tubular epithelial cell exfoliation (*P* < .001) and arteriolar hyalinosis (*P* = .002). After PSM, IgAN patients still had a higher ratio of global sclerosis (*P* < .001) and a lower ratio of tubular epithelial cell exfoliation (*P* = .008) compared to non-IgAN patients. There were no differences in the prevalence of tubular atrophy/interstitial fibrosis, and other vascular lesions, including fibrous necrosis, onion skin lesions, intravascular thrombosis, and intravascular erythrocyte fragments. Thus, there is a higher incidence of chronic pathological changes and a lower incidence of acute changes in mHTN-associated TMA patients with comorbid IgAN.

**Table 2:** Histopathologic findings of patients.

		Entire cohort			Propensity score-matched cohort		
Biopsy characteristics	Total (*n* = 292)	Non-IgAN (*n* = 206)	IgAN (*n* = 86)	*P* value	Non-IgAN (n = 61)	IgAN (n = 61)	*P* value
Global lesions, number							
Number of glomeruli	23 (18, 32)	25 (18, 34)	21 (16, 26.25)	.001	23 (16, 30)	21 (16, 27)	.167
Global sclerosis	8 (4, 13)	7 (4, 10.25)	12 (7, 19)	<.001	6 (3, 9)	11 (6, 17)	<.001
Segmental sclerosis	0.5 (0, 2)	0 (0, 1)	1 (0, 2)	.153	0 (0, 1)	1 (0, 2)	.344
Tubular lesions, *n* (%)							
Interstitial fibrosis and tubular atrophy				<.001			.246
<25%	10 (3.5)	9 (4.4)	1 (1.2)		3 (4.9)	1 (1.7)	
25% to <50%	57 (19.8)	48 (23.5)	9 (10.7)		10 (16.4)	8 (13.6)	
50% to <75%	173 (60.1)	125 (61.3)	48 (57.1)		39 (63.9)	33 (55.9)	
75% to 100%	48 (16.7)	22 (10.8)	26 (31.0)		9 (14.8)	17 (28.8)	
Tubular epithelial cell exfoliation	99 (33.9)	89 (43.2)	10 (11.6)	<.001	23 (37.7)	10 (16.4)	.008
Vascular lesions, *n* (%)							
Arteriolar hyalinosis	153 (52.4)	120 (58.3)	33 (38.4)	.002	31 (50.8)	25 (41.0)	.276
Onion skin lesions	175 (59.9)	129 (62.6)	46 (53.5)	.147	37 (60.7)	35 (57.4)	.713
fibrinoid necrosis	97 (33.2)	67 (32.5)	30 (34.9)	.696	31 (50.8)	24 (39.3)	.203
Intravascular thrombosis	52 (17.8)	34 (16.5)	18 (20.9)	.368	11 (18.0)	9 (14.8)	.625
Intravascular RBC fragments	29 (9.9)	19 (9.2)	10 (11.6)	.531	11 (18.0)	7 (11.5)	.307

RBC, red blood cell.

### Risk of concomitant IgAN on the primary outcome of kidney function recovery in patients with mHTN-associated TMA

**Figure 2:**
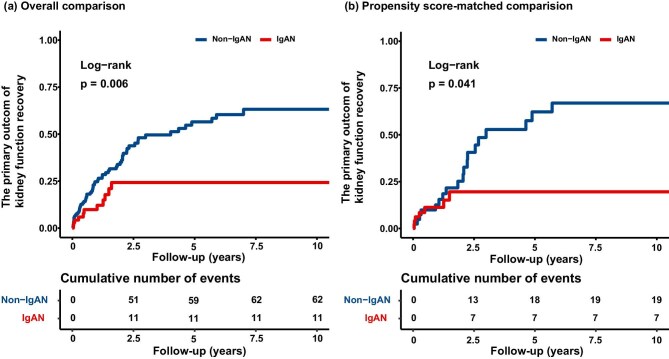
Cumulative incidence curve for a 50% decrease in creatinine, or a decrease in creatinine to normal, or kidney survival free from replacement therapy for 1 month, and free from dialysis for the patient. The log-rank test is used to compare the survival distributions between the groups, with the *P* value indicating the statistical significance of the observed differences in the rate of cumulative incidence: (**a**) overall comparison and (**b**) propensity score-matched comparison.

With a median follow-up period of 12.83 months (interquartile range 4.45–32.26), 73 (31.7%) of the primary outcomes occurred. The cumulative effect of patients with IgAN on the hazard of the occurrence of kidney function recovery was significantly lower compared to patients with non-IgAN (overall comparison, *P* = .006; propensity score-matched comparison, *P* = .041; Fig. 2). In the crude analysis, patients with concomitant IgAN were significantly associated with impaired kidney function recovery than those with non-IgAN (HR, 0.42; 95% CI, 0.22–0.79; *P* = .008). This difference remained statistically significant after adjustment for both the overall comparison (HR, 0.48; 95% CI, 0.24–0.96; *P* = .038) and the propensity score-matched comparison (HR, 0.41; 95% CI, 0.17–0.99; *P* = .047) (Table 3).

**Table 3:** Association between non-IgAN and IgAN and study outcome within the crude analysis, multivariable analysis, and propensity score analysis.

Variable	HR (95% CI) for study outcome	*P* value
The primary outcome of recovery of kidney function		
No. of events/no. of patients at risk (%)	73/230 (31.7)	<.001
Non-IgAN	62/159 (39.0)	
IgAN	11/71 (15.5)	
Crude analysis^a^	0.42 (0.22, 0.79)	.008
Multivariable analysis^b^	0.48 (0.24, 0.96)	.038
PSM^c^	0.41 (0.17, 0.99)	.047
The secondary outcome of KRT		
No. of events/no. of patients at risk (%)	115/245 (46.9)	<.001
Non-IgAN	68/170 (40.0)	
IgAN	47/75 (62.7)	
Crude analysis^a^	2.64 (1.79, 3.88)	<.001
Multivariable analysis^b^	2.31 (1.38, 3.88)	.002
PSM^c^	2.38 (1.14, 4.99)	.021

a The HRs from the bivariable model in all patients from the unmatched study.

b The HRs from the multivariable stratified Cox proportional hazards regression model, with additional covariate adjustment.

c The HR from propensity score-matched sample, constructed using a 1:1 nearest neighbor matching with a 0.02 caliper. The primary outcome was defined as a 50% decrease in creatinine, or a decrease in creatinine to normal, or kidney survival free from replacements therapy for 1 month. The secondary outcome was defined as starting KRT.

In addition, predictors for kidney function recovery in patients with mHTN-associated TMA were shown in Supplementary Table S1. In the multivariable Cox regression model adjusting for confounders with a *P* < .05 in the univariate regression analysis, IgAN was significantly associated with poorer kidney function recovery compared to patients with non-IgAN (adjusted HR, 0.42; 95% CI, 0.22–0.79; *P* = .008). Additionally, the results also indicated that higher level of platelet count (adjusted HR, 1.01; 95% CI, 1.01–1.01; *P* = .044) was related to an increased likelihood of kidney function recovery. The multivariable Cox regression analysis in the PSM cohort also indicated that patients with IgAN was an independent risk factor (adjusted HR, 0.27; 95% CI, 0.07–0.96; *P* = .043). After PSM, concomitant IgAN (HR, 0.41; 95% CI, 0.17–0.99; *P* = .047) was the only significant factor in the univariate Cox regression. Therefore, we included the factors with *P* < .05 in the overall comparison, commonly used drugs, and biopsy indicators in the multivariate Cox regression analysis. A comparable pattern was observed in the PSM cohort. After the multivariable Cox regression analysis, patients with IgAN (adjusted HR, 0.27; 95% CI, 0.07–0.96; *P* = .043) was identified as the only risk factor for the kidney function recovery in patients with mHTN-associated TMA (Table 4).

**Table 4:** Univariate and multivariable Cox regression analysis for the primary outcome of kidney function recovery in the PSM cohort.

Variables	Univariate HR (95%CI)	*P* value	Multivariable HR (95%CI)	*P* value
IgAN, (yes/no)	0.41 (0.17, 0.99)	.047	0.27 (0.07, 0.96)	.043
Hemoglobin, g/l	1.00 (0.98, 1.01)	.722		
Blood platelets count, 10^9^/l	1.00 (1.00, 1.01)	.347	1.00 (1.00, 1.01)	.762
Serum albumin, g/l	0.99 (0.92, 1.06)	.709		
Serum creatinine, mg/dl	1.00 (1.00, 1.00)	.540		
eGFR, ml/min/1.73 m^2^	0.99 (0.96, 1.03)	.755		
24-hour proteinuria, g/day	0.87 (0.60, 1.25)	.451	1.04 (0.69, 1.56)	.853
Complement 3, g/l	0.64 (0.07, 6.01)	.699		
Complement 4, g/l	0.61 (0.01, 53.19)	.829		
ACEI/ARBs, (yes/no)	1.11 (0.48, 2.56)	.812	1.49 (0.47, 4.69)	.498
β-blocker (yes/no)	1.08 (0.37, 3.13)	.892		
α-blocker (yes/no)	1.43 (0.62, 3.30)	.407		
Sacubitril/valsartan (yes/no)	1.00 (0.34, 2.96)	.995	1.95 (0.39, 9.69)	.414
Kidney pathology characteristic				
Segmental sclerosis, number	1.14 (0.90, 1.44)	.289	1.12 (0.85, 1.46)	.432
Tubular atrophy/interstitial fibrosis, *n* (%)				
<25%	1 (ref)		1 (ref)	
25% to <100%	0.86 (0.11, 6.43)	.881	0.85 (0.08, 9.17)	.894
Arteriolar hyalinosis, *n* (%)	0.76 (0.35, 1.65)	.490	0.36 (0.12, 1.07)	.066
Onion skin lesions, *n* (%)	0.91 (0.41, 2.01)	.819	0.63 (0.22, 1.82)	.396
fibrinoid necrosis, *n* (%)	1.26 (0.58, 2.73)	.559	0.90 (0.31, 2.64)	.847

### Risk of concomitant IgAN on the secondary outcome of kidney replacement therapy in patients with mHTN-associated TMA

**Figure 3:**
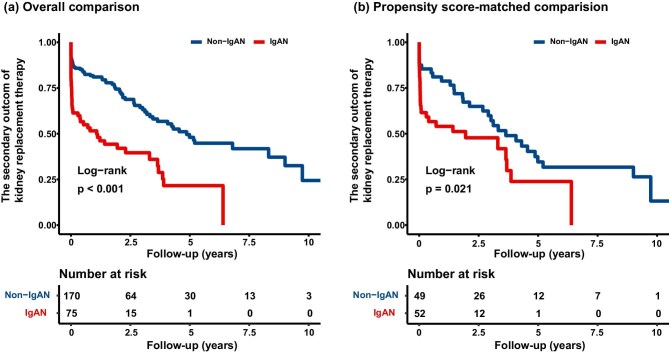
Survival curve for KRT in mHTN-associated TMA. The log-rank test is used to compare the survival distributions between the groups, with the *P* value indicating the statistical significance of the observed differences in the rate of progression to KRT: (**a**) overall comparison and (**b**) propensity score-matched comparison.

During the follow-up from 2008 to 2023, with a median follow-up period of 17.50 months (interquartile range: 2.17–37.83), 115 (46.9%) patients progressed to the secondary outcome of kidney replacement therapy. Patients with concomitant IgAN showed poorer outcomes of KRT than patients with non-IgAN (overall comparison, *P* < .001; propensity score-matched comparison, *P* = .021; Fig. 3). In the crude analysis, patients with IgAN exhibited a higher risk of KRT compared to non-IgAN patients (HR, 2.64; 95% CI, 1.79–3.88; *P* < .001). This difference remained statistically significant after adjustment for both the overall comparison (HR, 2.31; 95% CI, 1.38–3.88; *P* = .002) and the propensity score-matched comparison (HR, 2.38; 95% CI, 1.14–4.99; *P* = .021) (Table 3).

Risk factors for KRT in patients with mHTN-associated TMA are shown in Supplementary Table S2. In the multivariable Cox regression analysis, patients with IgAN were more likely to require KRT compared to non-IgAN patients (adjusted HR, 2.31; 95% CI, 1.38–3.88; *P* = .002). Furthermore, lower eGFR (adjusted HR, 0.93; 95% CI, 0.87–0.98; *P* = .014), higher serum creatinine (adjusted HR, 1.01; 95% CI, 1.01–1.01; *P* < .001), and higher 24-hour proteinuria (adjusted HR, 1.19; 95% CI, 1.06–1.34; *P* = .003) were associated with an increased risk of KRT. Multivariable Cox regression analysis in the PSM cohort also indicated that concomitant IgAN (adjusted HR, 2.38; 95% CI, 1.14–4.99; *P* = .021), higher serum creatinine (adjusted HR, 1.01; 95% CI, 1.01–1.01; *P* = .004), and without ACEI/ARBs treatment (adjusted HR, 0.49; 95% CI, 0.28–0.87; *P* = .015) were risk factors for the KRT in mHTN patients with TMA (Table 5).

**Table 5:** Univariate and multivariable Cox regression analysis for the secondary outcome of KRT in the PSM cohort.

Characteristics	Univariate HR (95%CI)	*P* value	Multivariable HR (95%CI)	*P* value
IgAN (yes/no)	1.87 (1.09, 3.22)	.023	2.38 (1.14, 4.99)	.021
Hemoglobin, g/l	0.97 (0.96, 0.98)	<.001	0.99 (0.98, 1.01)	.464
Blood platelets count, 10^9^/l	1.00 (0.99, 1.00)	.238		
Serum albumin, g/l	0.90 (0.86, 0.95)	<.001	0.96 (0.92, 1.01)	.136
Serum creatinine, mg/dl	1.01 (1.01, 1.01)	<.001	1.01 (1.01, 1.01)	.004
eGFR, ml/min/1.73 m^2^	0.91 (0.87, 0.95)	<.001	0.97 (0.92, 1.03)	.381
24-hour proteinuria, g/day	1.24 (1.06, 1.46)	.007	1.10 (0.89, 1.35)	.366
Complement 3, g/l	0.71 (0.18, 2.76)	.622		
Complement 4, g/l	2.28 (0.20, 26.20)	.509		
ACEI/ARBs, (yes/no)	0.56 (0.33, 0.94)	.027	0.49 (0.28, 0.87)	.015
β-blocker, (yes/no)	0.99 (0.52, 1.88)	.978		
α-blocker, (yes/no)	0.85 (0.51, 1.43)	.550		
Sacubitril/valsartan, (yes/no)	1.92 (1.00, 3.69)	.050		
Kidney pathology characteristic				
Segmental sclerosis, number	0.97 (0.80, 1.17)	.730		
Tubular atrophy/interstitial fibrosis, *n* (%)				
<25%	1 (ref)			
25 to <100%	3.17 (0.44, 22.90)	.253		
Arteriolar hyalinosis, *n* (%)	0.63 (0.37, 1.05)	.078		
Onion skin lesions, *n* (%)	1.10 (0.65, 1.86)	.710		
fibrinoid necrosis, *n* (%)	1.08 (0.64, 1.82)	.773		

### Intensive blood pressure control management

The study divided patients into two groups based on their discharge SBP into two groups, including those with SBP ≤130 mmHg and those with SBP >130 mmHg, to assess the impact of intensive blood pressure control. We assessed how the presence of IgAN affects the long-term outlook for kidney health under intensive antihypertensive therapy (Table 6). IgAN patients had a higher incidence of receiving KRT compared to the non-IgAN patients, regardless of the degree of blood pressure (*P* = .032). In addition, the non-IgAN patients were more conducive to the kidney function recovery of a 25% reduction in serum creatinine levels, regardless of the degree of blood pressure (*P* < .001). Importantly, non-IgAN patients were more likely to achieve the kidney function recovery of a 50% reduction in serum creatinine levels (*P* = .002) despite the absence of intensive blood pressure control.

**Table 6:** Estimated event rates among intensified antihypertensive therapy with and without IgAN.

	SBP ≤130 mmHg			SBP >130 mmHg		
Events	Non-IgAN	IgAN	*P* value	Non-IgAN	IgAN	*P* value
KD	36 (54.5)	23 (62.6)	.453	88 (62.9)	35 (71.4)	.279
KRT	18 (31.6)	18 (54.5)	.032	50 (44.2)	29 (69.0)	.006
Recovery 1 *	40 (71.4)	15 (45.5)	.015	67 (63.2)	11 (28.9)	<.001
Recovery 2 *	20 (35.7)	6 (18.2)	.079	42 (40.8)	5 (13.2)	.002
Recovery 3 *	5 (23.8)	2 (11.1)	.303	19 (32.8)	4 (13.3)	.049
Recovery 4 *	22 (47.8)	8 (38.1)	.457	42 (50.6)	5 (31.3)	.156

Recovery 1 *: A 25% decrease in creatinine, or a decrease in creatinine to normal, or kidney survival free from replacement therapy for 1 month.

Recovery 2 *: A 50% decrease in creatinine, or a decrease in creatinine to normal, or kidney survival free from replacement therapy for 1 month.

Recovery 3 *: Free from dialysis for patients dependent on dialysis at baseline.

Recovery 4 *: 15% increase in the eGFR.

KD, kidney failure.

## DISCUSSION

This observational cohort study found that mHTN-associated TMA patients with concomitant IgAN had significantly lower serum albumin, higher 24-hour proteinuria, lower tubular epithelial cell exfoliation, and higher prevalence of global sclerosis and segmental sclerosis in kidney biopsy specimens compared to patients with non-IgAN. The IgAN group also exhibited a lower usage of α-blockers and a higher usage of sacubitril/valsartan. Furthermore, IgAN patients were more likely to experience poorer kidney function recovery (overall comparison, HR, 0.48; 95% CI, 0.24–0.96; *P* = .038; propensity score-matched comparison, HR, 0.41; 95% CI, 0.17–0.99; *P* = .047) and progress to KRT outcomes (overall comparison, HR, 2.31; 95% CI, 1.38–3.88; *P* = .002; propensity score-matched comparison, HR, 2.38; 95% CI, 1.14–4.99; *P* = .021). Moreover, our study confirmed that concomitant IgAN is a significant risk factor for higher rates of KRT and poorer kidney function recovery, regardless of intensive blood pressure control in mHTN-associated TMA patients.

mHTN often leads to significant kidney complications, and the presence of coexisting IgAN may further worsen kidney outcomes. Patients with mHTN often experience kidney involvement, typically presenting with elevated serum creatinine levels, proteinuria, thrombocytopenia, and kidney failure, which are key markers of TMA [21]. In our cohort, we observed that some patients with mHTN-associated TMA also had concurrent IgAN, and this group was more likely to present with higher 24-hour proteinuria levels and lower serum albumin levels. The mechanism of this association may be attributed to IgAN promoting inflammatory responses and vascular damage [10, 22].

Understanding the pathology changes of mHTN-associated TMA is crucial [23, 24]. The primary lesions of TMA are usually concentrated in the kidney arterioles, with pathological features such as fragmented red blood cells in the lumen, onion skin changes in the kidney arterioles, arteriolar fibrinoid necrosis, and intraluminal thrombosis being the main pathological features in mHTN-associated TMA patients [7]. Throughout our study, it was consistently noted that most mHTN-associated TMA patients displayed heightened indicators of kidney vascular damage, such as global glomerulosclerosis, arteriolar hyalinosis, and onion skin lesions. We also found that the IgAN group exhibited a higher proportion of sclerotic glomeruli and more severe interstitial fibrosis and tubular atrophy. Similarly, a France study based on 128 patients with kidney biopsies found that the group with TMA had a significantly greater percentage of sclerotic glomeruli and worse tubulointerstitial fibrosis than those of the group without TMA in IgAN patients [25]. This suggests a mutual exacerbation of glomerular and interstitial damage between IgAN and TMA. In addition, we surprisingly found that mHTN-associated TMA patients coexisting IgAN showed less tubular epithelial cell exfoliation. Tubular epithelial cell exfoliation is often indicative of acute kidney injury [26], serving as a marker of the acute course of mHTN. Therefore, the lower level of tubular epithelial cell exfoliation in the IgAN group may be explained by the chronic nature of IgAN, where gradual immune-mediated damage dominates, in contrast to the acute endothelial injury typically observed in mHTN-related TMA, likely resulting in less severe tubular cell detachment.

While Sevillano *et al.* [27] confirmed hypertension, as a complication of IgAN, is a risk factor for progressive kidney function decline, the long-term kidney outcomes in mHTN patients with coexisting IgAN remain unclear. Therefore, we investigated the clinical features and kidney prognosis based on whether patients had concurrent IgAN or not. Our cohort study suggests that the presence of IgAN is associated with poor kidney prognosis in patients with mHTN-associated TMA. The IgAN group exhibited a lower incidence of kidney function recovery and a higher requirement for KRT. A potential explanation for the impaired kidney function recovery in the IgAN group could be the combined effects of chronic immune-mediated damage in IgAN and acute endothelial injury in mHTN, creating a ‘double-hit’ to kidney function. Furthermore, we observed more severe glomerulosclerosis and greater interstitial fibrosis and tubular atrophy in patients with coexisting IgAN. A multicenter study [28] from Egypt identified a higher number of sclerotic glomeruli and a larger interstitial fibrosis and tubular atrophy area as risk factors for end stage kidney disease, partially explaining the increased KRT demand in the IgAN group within our cohort of mHTN-related TMA patients. Similarly, a large UK IgAN cohort [29] reported that 50% of patients progressed to kidney failure or death within a median follow-up of 5.9 years. Our study further substantiates that, even in patients already suffering from mHTN kidney injury, the coexistence of IgAN continues to have a pronounced negative impact on kidney outcomes. Our cohort provides robust evidence that the presence of IgAN is an independent risk factor for poor prognosis, with the likelihood of kidney function recovery being only 0.27 times that of the non-IgAN group and a 2.38-fold increased risk of requiring KRT. Therefore, the coexistence of IgAN can be considered a key and readily identifiable predictor of poor outcomes in patients with mHTN-associated TMA.

The role of platelets in kidney diseases has been a focal point of research [30, 31]. Drolma Gomchok *et al.* [31] discovered that elevated platelet counts are associated with increased inflammation and endothelial dysfunction, both of which are critical factors in the progression of kidney diseases. Furthermore, a retrospective study by Jiaxing Tan *et al.* [32] has demonstrated that the platelet-to-albumin ratio is an independent risk factor for poor kidney prognosis in IgAN. Interestingly, our study's findings suggest that elevated platelet levels are a protective factor for kidney function recovery in patients with mHTN-associated TMA. This might be related to the anti-inflammatory effects of platelets by regulating macrophage functions, regulatory T cells, and secretion of proresolving mediators [30].

The choice of antihypertensive medication in mHTN-associated TMA is crucial for disease progression and kidney recovery [33, 34]. ACEI/ARBs, as classic antihypertensive agents, have been demonstrated to confer significant cardio-kidney benefits, particularly in patients with CKD [35]. In a long-term cohort study of IgAN by Kensuke A [34], the ACEI/ARB treatment group had the highest 20-year kidney survival rate. In addition, ACE/ARB treatment has been shown to reduce proteinuria and slow the progression of kidney disease and should be considered in conjunction with intensified antihypertensive strategies [36]. In our cohort, >60% of patients with mHTN-associated TMA were prescribed ACEI/ARBs. Patients receiving ACEI/ARBs had a 0.49-fold reduced risk of requiring KRT compared to those not on these medications, also reflecting the kidney protective effects of ACEI/ARBs.

Effective blood pressure control is essential for disease management and significantly influences quality of life [37, 38]. Intensive blood pressure control strategies, aimed at significantly reducing cardiovascular risks by targeting SBP levels below conventional thresholds, have emerged as a pivotal approach in hypertension management [39–41]. However, there have been no studies reported on how effective blood pressure control correlates with kidney prognosis in patients with mHTN-associated TMA. Our results show that even in patients with SBP ≤130 mmHg at discharge, those with IgAN are still more likely to start KRT and more challenging to achieve improvement in kidney function. Although this conclusion seems to be contrary to previous studies [36, 42], it emphasizes the importance of combined IgAN in kidney prognosis. Further research is necessary to increase the sample size and conduct more detailed blood pressure classifications to determine the optimal discharge blood pressure range for patients with mHTN-associated TMA.

### Limitations

Although our study yielded positive outcomes, it is important to recognize that there are several possible constraints that must be considered. First, this study was limited to Chinese patients with mHTN-associated TMA, so caution should be exercised before applying the conclusions of this study to patients with other ethnic backgrounds. Second, a comprehensive classification system for diverse TMA etiologies was not available in all patients with TMA confirmed by kidney biopsy in this study. A diagnosis of mHTN- associated TMA was made only after ruling out other secondary TMA causes. Third, the omission of the Oxford classification variable in the study for patients with IgAN may have introduced bias into the prognostic analysis. Finally, although we used PSM to reduce selection bias, some unmeasured confounding factors may still exist.

## CONCLUSIONS

In this cohort study, our study added to the accumulating evidence supporting that the comorbidity of IgAN contributed to a poorer long-term kidney outcome compared to non-IgAN patients with mHTN-associated TMA. The findings suggested that in terms of kidney recovery, monitoring pathological characteristics can facilitate early management and risk assessment.

## Supplementary Material

sfaf017_Supplemental_File

## Data Availability

The data underlying this article cannot be shared publicly to protect the study participants’ privacy. The datasets generated and analyzed during the current study are available from the corresponding author upon request.
