# Supplementary material for: Kidney outcomes of malignant hypertension-associated thrombotic microangiopathy in patients with and without IgA nephropathy: a propensity score-matched analysis
Source: Clin Kidney J. 2025 Feb 21;18(3):sfaf017. doi: 10.1093/ckj/sfaf017 (PMC11914877; doi:10.1093/ckj/sfaf017)
Supplement: sfaf017_Supplemental_File [file sfaf017_supplemental_file.docx]

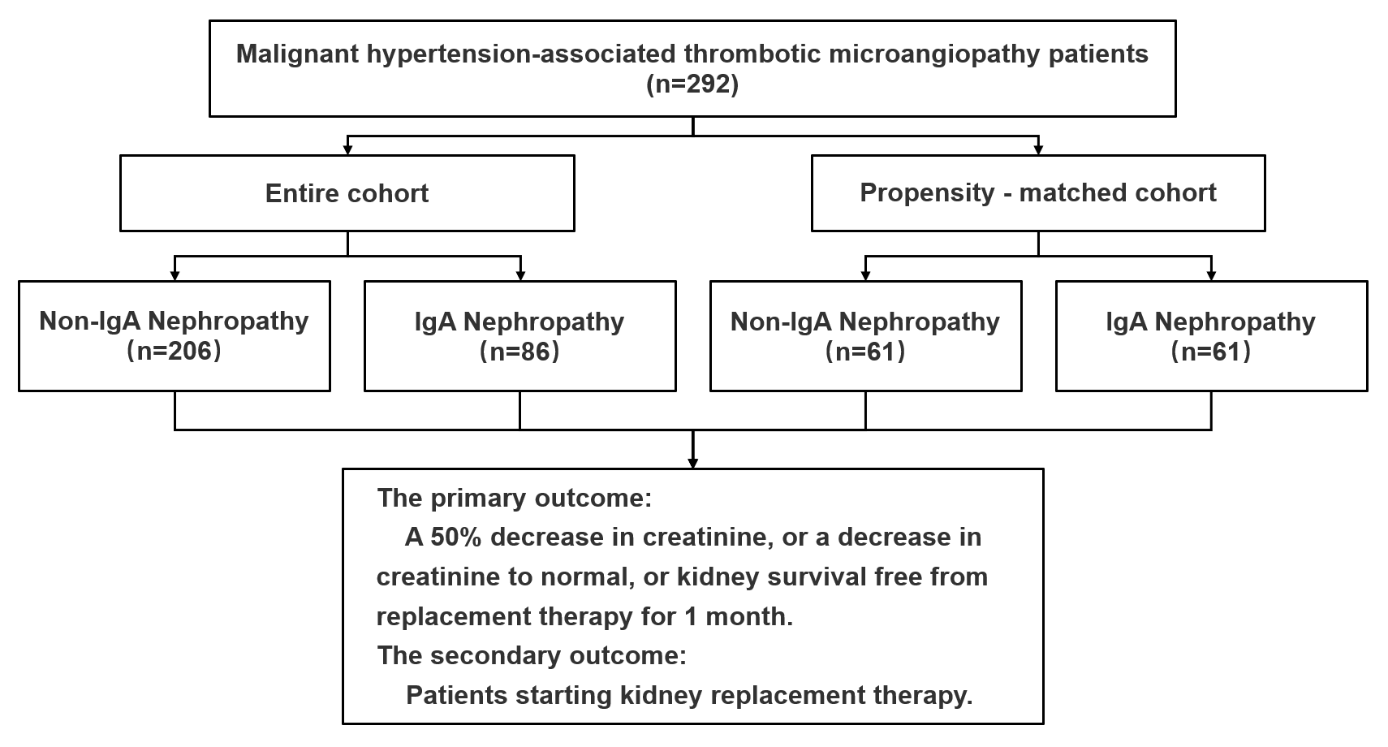
**Supplementary materials**

**Supplementary Figure S1. Flowchart of study participants.**

**Supplementary Table S1. Univariable and multivariable Cox regression analysis for the primary outcome of kidney function recovery in the entire cohort.**

| **Characteristics** | **Univariate HR (95%CI)** | ***P* value** | **Multivariable HR (95%CI)** | ***P* value** |
| --- | --- | --- | --- | --- |
| IgAN, (yes/no) | 0.42 (0.22, 0.79) | 0.008 | 0.48 (0.24, 0.96) | 0.038 |
| Hemoglobin, g/L | 1.00 (0.99, 1.01) | 0.909 | -- | -- |
| Blood platelets count, 10^9^/L | 1.01 (1.01, 1.01) | 0.008 | 1.01 (1.01, 1.01) | 0.044 |
| Serum albumin, g/L | 0.98 (0.94, 1.03) | 0.483 | -- | -- |
| Serum creatinine, mg/dL | 1.00 (1.00, 1.00) | 0.475 | -- | -- |
| eGFR, ml/min/1.73m^2^ | 0.98 (0.96, 1.01) | 0.193 | -- | -- |
| 24-hour proteinuria, g/day | 0.80 (0.65, 0.98) | 0.030 | 0.86 (0.71, 1.05) | 0.143 |
| Complement 3, g/L | 1.88 (0.68, 5.23) | 0.224 | -- | -- |
| Complement 4, g/L | 3.43 (0.39, 30.15) | 0.267 | -- | -- |
| ACEI/ARBs, (yes/no) | 0.91 (0.56, 1.49) | 0.715 | -- | -- |
| β-blocker, (yes/no) | 1.11 (0.55, 2.23) | 0.766 | -- | -- |
| α-blocker, (yes/no) | 1.19 (0.73, 1.95) | 0.477 | -- | -- |
| Sacubitril/valsartan, (yes/no) | 1.78 (1.05, 3.02) | 0.033 | 1.67 (0.95, 2.95) | 0.076 |
| **Kidney pathology characteristic** |  |  |  |  |
| Segmental sclerosis, number | 1.09 (0.93, 1.28) | 0.290 | -- | -- |
| Tubular atrophy/interstitial fibrosis, n (%) |  |  |  |  |
| <25% | 1 (ref) |  | -- | -- |
| 25 to <100% | 0.66 (0.21, 2.10) | 0.482 | -- | -- |
| Arteriolar hyalinosis, n (%) | 0.81 (0.51, 1.28) | 0.367 | -- | -- |
| Onion skin lesions, n (%) | 1.16 (0.71, 1.88) | 0.548 | -- | -- |
| fibrinoid necrosis, n (%) | 1.47 (0.92, 2.35) | 0.108 | -- | -- |

HR, hazard ratio; eGFR, estimated glomerular filtration rate; ACEI, angiotensin-converting enzyme inhibitor; ARBs, angiotensin II receptor blockers.

**Supplementary Table S2. Univariable and multivariable Cox regression analysis for the secondary outcome of kidney replacement therapy in the entire cohort.**

| **Characteristics** | **Univariate HR (95%CI)** | ***P* value** | **Multivariable HR (95%CI)** | ***P* value** |
| --- | --- | --- | --- | --- |
| IgAN, (yes/no) | 2.64 (1.79, 3.88) | < 0.001 | 2.31 (1.38, 3.88) | 0.002 |
| Hemoglobin, g/L | 0.97 (0.96, 0.98) | < 0.001 | 1.00 (0.98, 1.01) | 0.681 |
| Blood platelets count, 10^9^/L | 0.99 (0.99, 0.99) | 0.002 | 1.00 (0.99, 1.00) | 0.117 |
| Serum albumin, g/L | 0.91 (0.87, 0.94) | < 0.001 | 0.96 (0.93, 1.00) | 0.068 |
| Serum creatinine, mg/dL | 1.01 (1.01, 1.01) | < 0.001 | 1.01 (1.01, 1.01) | < 0.001 |
| eGFR, ml/min/1.73m^2^ | 0.88 (0.85, 0.91) | < 0.001 | 0.93 (0.87, 0.98) | 0.014 |
| 24-hour proteinuria, g/day | 1.24 (1.13, 1.35) | < 0.001 | 1.19 (1.06, 1.34) | 0.003 |
| Complement 3, g/L | 0.26 (0.10, 0.67) | 0.005 | 0.84 (0.26, 2.73) | 0.778 |
| Complement 4, g/L | 0.29 (0.05, 1.83) | 0.187 | -- | -- |
| ACEI/ARBs, (yes/no) | 0.57 (0.40, 0.83) | 0.003 | 0.71 (0.46, 1.09) | 0.115 |
| β-blocker, (yes/no) | 0.8 (0.49, 1.31) | 0.372 | -- | -- |
| α-blocker, (yes/no) | 1.03 (0.70, 1.51) | 0.894 | -- | -- |
| Sacubitril/valsartan, (yes/no) | 1.08 (0.66, 1.75) | 0.770 | -- | -- |
| **Kidney pathology characteristic** |  |  |  |  |
| Segmental sclerosis, number | 0.94 (0.81, 1.10) | 0.470 | -- | -- |
| Tubular atrophy/interstitial fibrosis, n (%) |  |  |  |  |
| <25% | 1 (ref) |  | -- | -- |
| 25 to <100% | 6.13 (0.86, 43.92) | 0.071 | -- | -- |
| Arteriolar hyalinosis, n (%) | 0.67 (0.46, 0.97) | 0.032 | 1.31 (0.84, 2.05) | 0.235 |
| Onion skin lesions, n (%) | 0.94 (0.65, 1.36) | 0.738 | -- | -- |
| fibrinoid necrosis, n (%) | 0.96 (0.65, 1.42) | 0.851 | -- | -- |

HR, hazard ratio; eGFR, Estimated glomerular filtration rate; ACEI, angiotensin-converting enzyme inhibitor; ARBs, angiotensin II receptor blockers.
